# Protective Effects of Gelam Honey against Oxidative Damage in Young and Aged Rats

**DOI:** 10.1155/2014/673628

**Published:** 2014-11-19

**Authors:** Zulaikha Sahhugi, Siti Maisarah Hasenan, Zakiah Jubri

**Affiliations:** Department of Biochemistry, Faculty of Medicine, The National University of Malaysia, Jalan Raja Muda Abdul Aziz, 50300 Kuala Lumpur, Malaysia

## Abstract

Aging is characterized by progressive decline in physiological and body function due to increase in oxidative damage. Gelam honey has been accounted to have high phenolic and nonphenolic content to attenuate oxidative damage. This study was to determine the effect of local gelam honey on oxidative damage of aged rats. Twenty-four male Spraque-Dawley rats were divided into young (2 months) and aged (19 months) groups. Each group was further divided into control (fed with plain water) and supplemented with 2.5 mg/kg body weight of gelam honey for 8 months. DNA damage level was determined by comet assay and plasma malondialdehyde (MDA) by high performance liquid chromatography (HPLC). The activity of blood and cardiac antioxidant enzymes was determined by spectrophotometer. The DNA damage and MDA level were reduced in both gelam honey supplemented groups. Gelam honey increases erythrocytes CAT and cardiac SOD activities in young and cardiac CAT activity in young and aged groups. The DNA damage was increased in the aged group compared to young group, but reduced at the end of the study. The decline of oxidative damage in rats supplemented with gelam honey might be through the modulation of antioxidant enzyme activities.

## 1. Introduction

Aging is a decline process in physiological function that occurs gradually and continuously. It affects homeostasis process and increases the tendency to develop disease and eventually death [1]. Aging is a multifactorial process which can be found out by genetic or/and influenced by environmental factors [2]. Many theories of aging have been postulated, but the most famous one is the free radical theory proposed by Harman [3]. The damages of macromolecules by reactive oxygen species (ROS) cause irreversible cell damage and lead to cell dysfunction. ROS are mainly generated in uncompleted cellular respiration in mitochondria [4]. The accumulation of mutation and deletion of mitochondrial DNA (mtDNA) may lead to DNA damage [5] and resulted in an improper function of electron transport chain (ETC) [6]. The production of ROS also has related to life span [7]. Thus, the consumption of antioxidant will help to slow down the aging process by preserving the cells to act normally and increase the life span.

An antioxidant that was found in natural products such as herbs, medicinal plants, spices, and honey can act as a ROS scavenger and prevent cells and tissues from oxidative damage [8]. There are several local honeys such as nenas, coconut, borneo tropika honeys, and tualang honey. In this study, we focused on gelam honey because previous study reported that gelam honey has high flavonoids and phenolic contents that has an antioxidative effect as compared to other local honeys [9, 10]. Studies claimed that phenolic composition and antioxidant content of honey are depending on the source of the flowers, season, and environmental factors [11]. Studies have reported that gelam honey has other effects such as anti-inflammatory response [12], increase of the healing process [13], and being a radioprotectant agent [14].

Honey is well-known for its medicinal and health promoting characteristics [15]. Honey is carbohydrate-rich syrup produced by honey bees from the nectar of flowers and secretion of other plants [16]. Honey consists of carbohydrates, free amino acids, vitamins, trace elements flavonoids, and phenolic compounds [17]. Previous studies reported that the major phenolics compounds of gelam honey are caffeic acid gallic acid, ferulic acid chlorogenic acid, p-coumaric acid, ellagic acid, quercetin, chrysin, and hesperetin [18].

## 2. Materials and Methods

### 2.1. Gelam Honey

Gelam honey was obtained from the Department of Agriculture, Batu Pahat, Johor, Malaysia. Gelam honey was irradiated at the dosage of 25 kGy using ELORADO-8 with Cobalt-60 as its source for sterilization at Malaysian Nuclear Agency. The irradiated honey was kept in the dark place at room temperature.

### 2.2. Experimental Animals

A total of 24 male Sprague-Dawley rats aged 2 months and 19 months were divided into two groups each consisting of control (*n* = 6) and gelam honey (*n* = 6) group. The rats were obtained from Laboratory Animal Resource Unit, Faculty of Medicine, The National University of Malaysia. The experimental protocol was approved by The National University of Malaysia Animal Ethics Committee (FB/BIOK/2011/ZAKIAH/21-SEPTEMBER/388-SEPTEMBER-2011-DECEMBER-2012). The control group was force-fed with water 2.5 mL/kg body weight while treatment group was supplemented with 2.5 mg/kg body weight of gelam for 8 months. The dose used for gelam honey is equal to 1 teaspoon of honey consumed by Malaysian. The rats were fed with commercial rat pellets (Gold Chain, Malaysia) and plain water ad libitum.

### 2.3. Sample Preparation

#### 2.3.1. Blood

Blood was withdrawn at 0 months (before the treatment), 4 months, and 8 months. The rats were anaesthetized with Zoletil (Virbac, France) before blood withdrawn via orbital sinus. Approximately 6 mL of blood was collected using capillary tube and was put into the heparin (Leo, Denmark) tube. The blood was kept in the ice promptly. Small amount of fresh whole blood was used for comet assay and the rest was centrifuged at 3000 rpm, 4°C for 10 min to obtain plasma and red blood cells. The plasma obtained was divided into aliquots and stored at −80°C for MDA assay. Erythrocytes were washed three times with normal saline, separated into aliquots, and stored at −80°C for antioxidant enzymes assays.

#### 2.3.2. Tissue Homogenate

After 8 months of treatment, rats were killed and hearts were excised out. The heart tissue was washed with ice-cold 1.15% NaCl (Sigma, St Louis, USA) (pH 7.2). The tissue was weighed about 100–200 mg before minced into smaller pieces. The tissue was homogenized using Ultra Turrax T25 Homogenizer (IKA Labortechnik, Germany). The homogenate was then centrifuged at 600 g for 10 min at 4°C using Bench Top Refrigerated Centrifuge Sorvall RC-5B. The supernatant was taken and centrifuged again at 12 000 g for 15 min at 4°C. The final supernatant was a cytosolic fraction. The supernatant was collected and stored at −80°C for enzyme assays (SOD, CAT, GPx). All the procedures were carried out on ice.

### 2.4. Comet Assay

Comet assay is a simple, quick, and sensitive method to measure the fraction of the DNA strands. This method was based on Singh et al. [19] with slight modification. Fresh blood about 5 *μ*L was mixed with 0.6% low melting point agarose (LMA) (Sigma-Aldrich, USA) and rapidly pipetted onto 0.6% normal melting point agarose (NMA) (ICN Biomedicals, USA) layer and covered with a coverslip. The mix was solidified for 15 min. Then, the coverslip was removed and the slides were immersed in cold lysing solution for 1 hour in 4°C. After 1 hour, the slides were removed from lysing solution and were placed in a horizontal gel electrophoresis platform in the freshly prepared and cooled (1–10°C) electrophoresis buffer to the depth of approximately 0.25 cm. The slides were kept in the solution for 20 minutes. The electrophoresis was conducted at 1–10°C for 20 minutes using 25 V with the current being adjusted to 300 mA by a change of the buffer volume.

After electrophoresis, the slides were placed horizontally and neutralization buffer was dropped. The slides were allowed to stand for 5 min. This was done for 3 times. The slides were drained and 30 *μ*L EtBr was added to each slide. All the slides were placed in a humidified air-tight container in a refrigerator to prevent dying of the gel. Following that, slides were analyzed as soon as possible under 200x magnification using a fluorescence microscope (AxioCam MRC, Carl Zeiss, Germany). Scores assigned on an arbitrary scale of 0–4 were based on perceived comet tail length migration and relative proportion of DNA in the comet tail. Five hundred nonoverlapping cells were randomly selected on each slide by categorizing cells as undamaged cells without tail (type 0), cells with tiny cell (1), cell with a dim tail (type 2), cells with a clear type (type 3), and only tail (type 4).

Total damage score for each slide can be calculated by multiplying the number of cells assigned to each grade of damage by the numeric value of the grade and summing over all grades:

(1) Arbitrary  unit=score  0×(N)+score  1(N) +score  2(N)+score  3(N)+score  4(N)N=the  number  of  cells assigned  to each  grade  of  damage.

### 2.5. Determination of Plasma MDA

Plasma malondialdehyde (MDA) was determined using high performance liquid chromatography (HPLC) with photodiode array detector (Shimadzu, Japan) as described by Pilz et al. [20] with some modifications. Briefly, samples (50 *μ*L) were mixed with 200 *μ*L of 1.3 M NaOH and incubated at 60°C for 30 min. After cooling the mixture, 100 *μ*L of 35% HCIO_4_ was added and centrifuged at 10 000 g for 10 min. Supernatant of the samples (300 *μ*L) was transferred into 1.5 mL of HPLC tubes and 5 mM of DNPH solution (50 *μ*L) was added into the mixture and incubated for 30 min at room temperature. Then, samples (40 *μ*L) were injected into the HPLC.

The amount of MDA was expressed as concentration of MDA in nmol per mL plasma.

### 2.6. Superoxide Dismutase Assay (SOD)

SOD was assayed using the method described by Beyer Jr. and Fridovich [21]. Briefly, 1.0 mL aliquots of a mixture containing 0.1 mM phosphate buffer pH 7.8, 57 *μ*M nitro blue tetrazolium (Sigma, St Louis, USA), 9.9 mM L-methionine (Sigma, St Louis, USA), and 0.025% Triton-X (Sigma, St Louis, USA) were pipetted into test tubes. Then, 20 *μ*L of lysate or cardiac cytosol and 10 *μ*L of a solution containing 4.4 mg/100 mL riboflavin (Sigma, St Louis, USA) were added into the mixture. The tubes were illuminated for 7 min in an aluminium foil-lined box containing two 20-W Sylvania GroLux fluorescent lamps. Absorbance was then measured at a wavelength of 560 nm. A stock hemolysate was prepared by adding an equal volume of distilled water. One unit of SOD was defined as the amount of enzyme required to inhibit nitro blue tetrazolium reduction by 50% per min per mL lysate or cytosol. Enzyme activity was expressed as units per mg of Hb or protein (U/mg Hb or mg protein).

### 2.7. Catalase (CAT)

CAT was assayed using the method described by Aebi [22]. The reaction mixture consisted of 50 mM phosphate buffer pH 7.0 and 30 mM hydrogen peroxide. A stock hemolysate containing 5 g Hb/100 mL was prepared by adding four parts by volume of distilled water to the sample. A 1 : 500 dilution of this concentrated hemolysate was prepared by adding 50 mM phosphate buffer pH 7.0 immediately before running the enzyme assay and 1 : 200 dilution for cardiac cytosol. Then, the reaction was started by adding 1 mL of 30 mM H_2_O_2_. The absorbance was measured at 240 nm. One unit of catalase enzyme was defined as the amount of enzyme which liberates half the peroxide oxygen from H_2_O_2_ solution in 30 s at room temperature. Enzyme activity was expressed as units per mg of Hb or protein (U/mg Hb or mg protein). Hemoglobin in the hemolysate was measured by using Eagle diagnostic kit (Japan). Protein in cardiac cytosol was determined using the Bradford method [23].

### 2.8. Glutathione Peroxidase (GPx)

Glutathione peroxidase was assayed using the method described by Paglia and Valentine [24]. The reaction mixture contained 0.05 M phosphate buffer pH 7.0, 8.4 mM NADPH (Sigma, St Louis, USA), 1.125 M sodium azide (Hopkin & William, England), 5 mM reduced glutathione (GSH), NADPH (Sigma, St Louis, USA), and 3 U/mL glutathione reductase (Sigma, St Louis, USA). The hemolysate was prepared by adding an equal volume of distilled water to the RBC pellet and allowed to stand for 1 h at 4°C. Then four parts by volume of distilled water were added. Finally, double strength Drabkin's reagent (Eagle Diagnostics, Japan) was added to yield the final hemolysate. The reaction was initiated by adding 0.1 mL of 2.2 mM H_2_O_2_ (Merck, Darmstadt, German). The conversion of NADPH to NADP^+^ was followed by measuring the change in O.D./min at 340 nm. One unit of GPx was defined as the amount of enzyme required to oxidize 1 *μ*mol NADPH/min per mL lysate or cardiac cytosol. Enzyme activity was expressed as milliunits per mg of Hb or protein (mU/mg Hb or mg protein).

### 2.9. Statistical Analyses

All data were expressed as mean ± SD (*n* = 6–8) and differences between groups were statistically analyzed by variance (ANOVA). Differences were considered to be statistically significant if *P* < 0.05. All statistical analyses were carried out using SPSS for Windows version 16.0.

## 3. Results

### 3.1. Weight Changes

**Figure 1:**
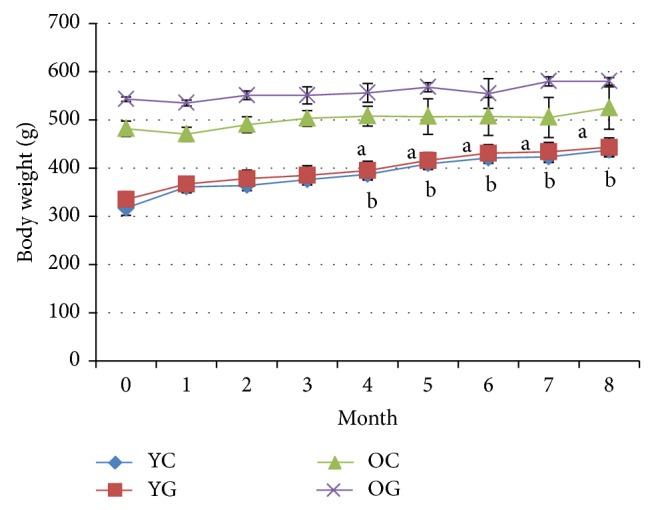
The effect of body weight changes in young (2 months) and old (19 months) rats with gelam honey supplementation for 8 months of treatment. Data are means ± SEM for 6 animals. a indicates a significant difference as compared to young control group at 0 months (*P* < 0.05), b indicates a significant difference compared to gelam group at 0 months (*P* < 0.05). YC indicates young control, YG indicates young gelam, OC indicates old control, and OG indicates old gelam.

As presented in Figure 1, the weight of young group was increased significantly from the baseline (0 months) after 4 months of treatment till the end of the study with or without gelam honey supplementation but no changes were observed in aged group.

### 3.2. Determination of Oxidative Damage

**Figure 2:**
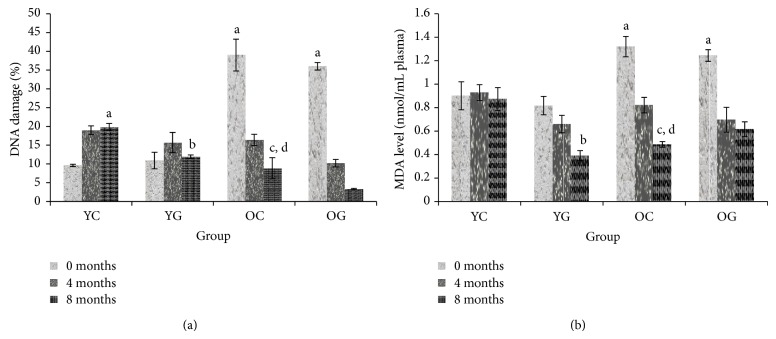
The effects of gelam honey supplementation on DNA damage (a) and plasma MDA level (b) of young (2 months) and old (19 months) groups. The results are expressed as mean ± SEM for 6 animals. a indicates significant difference compared to young control group at 0 months, b indicates significant difference compared to young control group at 8 months. c indicates significant difference compared to aged control group at 0 months. d indicates significant difference compared to young control group at 8 months. YC indicates young control, YG indicates young gelam, OC indicates old control, and OG indicates old gelam.

In the beginning of the study, DNA damage and plasma MDA level were increased in aged control group compared to young control group (Figure 2). At the end of the study, the DNA damage and plasma MDA level were reduced in aged control group compared to young control group. The reduction started at 4 months until the end of the study. Gelam honey supplementation reduces the DNA damage and plasma MDA levels in young but no changes were observed in aged group when compared to their respective control group.

### 3.3. Antioxidant Enzymes Activities

**Figure 3:**
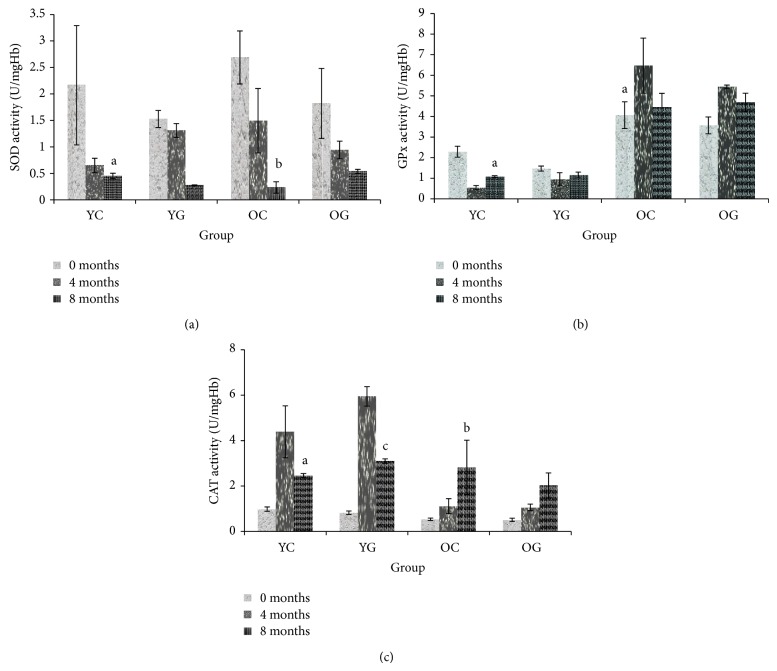
The effects of gelam honey supplementation on SOD (a), GPx (b), and CAT (c) of young (2 months) and old (19 months) groups. The results are expressed as mean ± SEM, (*P* < 0.05). a indicates significant difference compared to young control group at 0 months. b indicates significant difference compared to old control group at 0 months. c indicates significant difference compared to young control group at 8 months. YC indicates young control, YG indicates young gelam, OC indicates old control, and OG indicates old gelam.

At the baseline, the activity of SOD and CAT in the erythrocytes was found to be not changed in young group compared to aged group, but GPx activity was increased in aged group (Figure 3). At the end of the study, SOD activity was reduced in both young and aged groups. GPx activity was also reduced in young group and remains high in aged group, but CAT activity was increased in both age groups. Gelam honey supplementation does not change SOD and GPx activity in both age groups as compared to their respective control group but increased CAT activity in young group.

**Figure 4:**
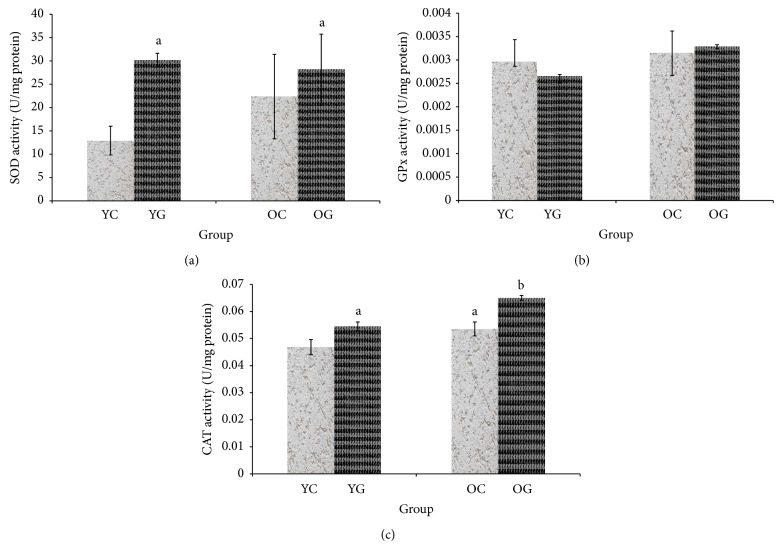
The effect of gelam honey supplementation on SOD (a), GPx (b), and CAT (c) activity of cardiac tissue in young and aged rats. The results are expressed as mean ± SEM (*P* < 0.05). a indicates significant difference compared to young control group. b indicates significant difference compared to aged control group. YC indicates young control, YG indicates young gelam, OC indicates old control, and OG indicates old gelam.

Cardiac CAT activity was increased in aged control group as compared to young control group. Gelam honey supplementation significantly increased cardiac SOD activity in young and cardiac CAT activity in both young and aged groups, but no changes were observed for GPx activity in both young and aged groups (Figure 4).

## 4. Discussion

The focus of the study is to determine the ability of gelam honey as a prevention agent against oxidative damage and thus resulted in slowing down the aging process. This is exceedingly important because oxidative damage can lead to aging and degenerative diseases such as cardiovascular disease, diabetes mellitus, and Alzheimer's disease. Honey could provide with invaluable nutritional ingredients and antioxidant substance and trace components, such as copper, zinc, and unidentified materials [25] that could ensure an elderly stay in healthy lives.

In this study, we used an irradiated gelam honey. Makpol et al. [14] reported that there was no significant difference in free radical-scavenging activity between irradiated and nonirradiated gelam honey but the radiation will kill microorganisms that may speed up 5-hydroxymethylfurfural (HMF) formation and keep honey to stay fresh and preserve its antibacterial activity [26]. Therefore, radiation is a crucial step in preserving the purity and quality of honey. During the supplementation period, honey must be kept at room temperature and put in the dark place to preserve its purity [27]. HMF is a cyclic aldehyde produced as a result of sugar degradation [28]. Belitz and Grosch [29] reported that the natural form of honey that contains simple sugars such as glucose, fructose, and many acids is favorable condition for the production of this substance.

Gelam honey supplementation caused no drastic body weight changes in this study. The changes observed were due to the amount of calorie intake. This was supported by Chepulis [30] who reported that honey supplementation did not increase the body weight of young and the aged rats. Swanson et al. [31] reported that total cholesterol and low density lipid cholesterol (LDL-C) in overweight healthy rats were reduced after honey supplementation. This might be due to the presence of substances in honey that could act together to prevent hyperlipidemia and cholesterol formation [32].

Oxidative damage was increased in aged rats at the beginning of the study but reduced at the end of the study. Harman [1] postulated free radical theory of aging process. The accumulation of free radicals will lead to oxidative damage and subsequently caused aging and degenerative diseases. In tendency to stabilize itself, free radicals attack macromolecules such as DNA, protein, and lipid. Damages to DNA will cause mutation and disturbance to the whole function of the cells. Lipid peroxidation may lead to membrane malfunction by impeding cell membrane fluidity and alters the activity of membrane-bound enzymes and receptors [33]. Lipid peroxidation of unsaturated fatty acids has been used as an indicator to measure increased oxidative stress and subsequently oxidative damage [34]. It supported our finding for aged control group at the beginning of the study and in young control group after 8 months of treatment. Study by Lopes et al. [35] on the colon mitochondria of aged rats (24–30 months) showed decrease of cristae and fragmentation of the mitochondrial membranes and an increase in smooth muscle cell apoptosis in aged animals. Mitochondria are a major site of ROS production (~90%). If mitochondria in aged group are damaged and undergo cell death, the production of ROS will decrease due to the lesser amount of functional mitochondria and resulted in the reduction of oxidative damage in aged group. Cells with high levels of mutant mtDNA will also have lower respiratory capacity and eventually resulted in mitochondrial dysfunction [36].

Gelam honey protection against oxidative damage might be better seen in young or middle aged group [37]. Furthermore, supplementation of nutrient high in antioxidants has been claimed to give therapeutic effects towards subjects with high oxidative stress [38]. Young to middle aged groups have shown protection against oxidative damage with gelam honey supplementation. It might be due to an optimal antioxidant defense system which is able to lower oxidative damage. Therefore, younger individuals are more able to encounter against oxidative stress compared to the older individuals [39].

Antioxidant enzymes are the first line of defense mechanism against oxidative damage [40]. The SOD catalyzes the dismutation of superoxide anion (O_2_^•−^) to hydrogen peroxide (H_2_O_2_) and oxygen (O_2_). Hydrogen peroxide will be converted into water by catalase and glutathione peroxidase. The net outcome of these two reactions is two potentially harmful species (superoxide and hydrogen peroxide) which are converted to water [41]. Our study showed no changes in SOD and CAT activity in erythrocytes of aged group compared to young group but the GPx activity was increased in aged group. At the end of the study, SOD and GPx activity were reduced but CAT activity was increased in both age groups. Antioxidant enzymes activity can be increased, decreased, or not changed with age [42, 43]. Gelam honey supplementation tends to further increase CAT activity in young group, but no changes were observed in the other antioxidant enzyme activity in both young and aged groups. Honey was proven to contain small amounts of catalase [44]. This possibly contributed to the increasing of CAT activity in the young rats.

In the cardiac tissue, only CAT activity increased in aged control group. This might be due to encounter effect of CAT activity towards the increased production of free radical in aging. This finding was supported by Inal et al. [45] showing that CAT activity was increased during aging in healthy aged group. Gelam honey increased cardiac SOD activity in young and cardiac CAT activity in both young and aged groups, but no changes were observed for GPx activity in both young and aged groups. Superoxide dismutase catalyzes the dismutation of superoxide into hydrogen peroxide and oxygen. The hydrogen peroxide then will be decomposed by CAT to water and oxygen [41]. Thus, the oxidative damage was reduced in young group with gelam honey supplementation. This is supported by Yao et al. [37] that found gelam honey reduces oxidative damage of young and middle aged rats by modulating antioxidant enzyme activities. Modulation in antioxidant enzyme activities in a different location will indicate a balance between enzymatic and nonenzymatic radical scavenger in scavenging, detoxify reactive oxygen species, and respond to oxidative stress in the whole body or certain organ such as in the cardiac tissue.

Gelam honey was demonstrated to be a good antioxidant both* in vitro* and* in vivo* [46]. Previous studies by Chua et al. [47] demonstrated that gelam honey has antioxidant and free radical scavenging activity which was correlated with its biochemical constituents such as total phenol, total flavonoid content, and total water-soluble vitamins (vitamin B_1_, B_2_, B_3_, B_9_, B_12_, and vitamin C), thus suggesting that gelam honey is capable of protecting against oxidative damage in aging. The exact mechanism by which gelam honey enhances the activity of antioxidant enzymes is not yet determined.

Honey has been used worldwide for many purposes such as for disease treatments [48]. Some of honey therapeutic effects on diseases underlie antioxidant mechanism such as anti-inflammatory [48], antibacterial activity [49], and wound healing [50]. Aljadi and Kamaruddin [9] reported that honey contains both aqueous and lipophilic antioxidants, which have an interaction with each other and subsequently make honey an ideal natural antioxidant by acting at different cellular sites. Phenolic content in honey plays a role as an antioxidant by reducing oxidative damage and has the ability to scavenge free radical activity [10]. Hussein et al. [51] reported that gelam honey contains gallic acid and ferulic acid that was not found in nenas honey. Gallic acid is one of the strongest free radical scavengers. It has a potential to decrease lipid peroxidation through divalent ions decrement which can act as catalyst towards lipid peroxidation process [52].

The modulation of antioxidant enzyme activity is more prominent in young rats compared to the aged rats even though both groups gave a reduction in DNA damage and lipid peroxidation level. The modulation of enzyme activity by gelam honey decreases with age. This might be contributed to the less prominent modulation of enzyme activity in the aged rats. Consequently, the supplementation is more effective with younger age than in older age.

## 5. Conclusions

Gelam honey reduced the oxidative damage through the modulation of antioxidant enzyme activity which was more prominent in young group compared to aged group. The action of honey to prevent oxidative damage might be due to its phenolic and nonphenolic antioxidant content that might trigger the modulation of antioxidant enzyme activity.
